# Prevalence of use of non-prescription analgesics in the Norwegian HUNT3 population: Impact of gender, age, exercise and prescription of opioids

**DOI:** 10.1186/s12889-015-1774-6

**Published:** 2015-05-02

**Authors:** Ola Dale, Petter C Borchgrevink, Olav Magnus S Fredheim, Milada Mahic, Pål Romundstad, Svetlana Skurtveit

**Affiliations:** Pain and Palliation Research Group, Department of Circulation and Medical Imaging, Faculty of Medicine, Norwegian University of Science and Technology, Trondheim, Norway; Department of Research and Innovation, St. Olav’s University Hospital, Trondheim, Norway; National Competence Centre for Complex Symptom Disorders, Department of Pain and Complex Disorders, St. Olav’s University Hospital, Trondheim, Norway; Centre of palliative medicine, Akershus University Hospital, Lorenskog, Oslo Norway; Department of Pharmacoepidemiology, Norwegian Institute of Public Health, Oslo, 0473 Norway; Department of Public Health and General Practice, Faculty of Medicine, Norwegian University of Science and Technology, Trondheim, Norway; Norwegian Centre of Addiction Research, University of Oslo, Oslo, Norway

**Keywords:** Non-prescription analgesics, Pain, General population, Opioids, Physical activity

## Abstract

**Background:**

There are concerns about potential increasing use of over-the-counter (OTC) analgesics. The aims of this study were to examine 1) the prevalence of self-reported use of OTC analgesics; 2) the prevalence of combining prescription analgesics drugs with OTC analgesics and 3) whether lifestyle factors such as physical activity were associated with prevalence of daily OTC analgesic use.

**Methods:**

Questionnaire data from the Nord–Trøndelag health study (HUNT3, 2006–08), which includes data from 40,000 adult respondents. The questionnaire included questions on use of OTC analgesics, socioeconomic conditions, health related behaviour, symptoms and diseases. Data were linked to individual data from the Norwegian Prescription Database. A logistic regression was used to investigate the association between different factors and daily use of paracetamol and/or non-steroid anti-inflammatory drugs (NSAIDs) in patients with and without chronic pain.

**Results:**

The prevalence of using OTC analgesics at least once per week in the last month was 47%. Prevalence of paracetamol use was almost 40%, compared to 19% and 8% for NSAIDs and acetylsalicylic acid (ASA), respectively. While the use of NSAIDs decreased and the use of ASA increased with age, paracetamol consumption was unaffected by age. Overall more women used OTC analgesics. About 3-5% of subjects using OTC analgesics appeared to combine these with the same analgesic on prescription. Among subjects reporting chronic pain the prevalence of OTC analgesic use was almost twice as high as among subjects without chronic pain. Subjects with little physical activity had 1.5-4 times greater risk of daily use of OTC compared to physically active subjects.

**Conclusions:**

Use of OTC analgesics is prevalent, related to chronic pain, female gender and physical inactivity.

## Background

In many countries, some analgesics are available over-the-counter (OTC) without a prescription from a doctor. Although OTCs are considered safe when used appropriately, intermittently and in the recommended doses, they have the same hazards regardless of source of supply. Not only is liver failure related to chronic unintended overdose of paracetamol, but the safety of chronic use of the drug was recently challenged [1]. Also non-steroid anti-inflammatory drugs (NSAIDs) and acetylsalicylic acid (ASA) pose gastrointestinal and cardiovascular hazards as well as risk of serious interactions with other drugs [2]. Therefore, the overall use of these drugs may have significance for the general health of a population, not least potential inappropriate use as indicated by concomitant use of prescribed drugs and OTC use of similar drugs [3].

Prevalence estimates for the use of OTC analgesics in previous studies varied between countries [3-6]. Many of these studies are 10–20 years old, different methodologies and case definitions have been applied. In a survey from 2003, treatment of chronic pain, including prescription and non-prescription analgesics, was investigated by interview questionnaire among long-lasting pain responders in 15 European countries. Considerable differences in prevalence of non-prescription medicines use between different countries were observed in spite of applying the same methodology across countries [7]. In a Norwegian population-based study from 1986–87, comprising almost 20,000 individuals, it was found that 28% of women and 13% of men had used analgesics in the past two weeks [5].

The following analgesics are available as OTC on the Norwegian market: paracetamol; NSAIDs: ibuprofen, naproxen and diclofenac; ASA and phenazone. Paracetamol is most widely used followed by ibuprofen. According to wholesale data use of OTC analgesics increased in Norway for 20 years until 2008, but has been stabilized last years [8].

Several factors have been shown to be associated with analgesic use in the general population [4,5,9,10]. Exercise is increasingly appreciated as an important factor that benefits general public health, also among individuals suffering from chronic pain [11], and therefore associations between exercise and OTC use should be examined together with other lifestyle and socio-demographic factors.

Analgesic OTC may constitute a health hazard to public health. Food and Drug Administration (FDA) has launched a campaign to advice on safe use of these drugs [12]. Research that discloses how analgesic OTC’s are used and by whom may provide valuable knowledge for such campaigns. In the present study, which is based on a major questionnaire-based population study in Nord–Trøndelag (HUNT3) linked to the complete national Norwegian Prescription Database (NorPD), the following specific questions were addressed:

1. What is the prevalence of self-reported use of the OTC group’s’ paracetamol, NSAIDs or ASA at least once a week in the last month?
  - Overall, and in men and women
  - With age
  - Daily in the last month for muscle/joint pain or headache
  - With chronic pain
  - With opioid use
2. What is the prevalence of prescribed analgesics 4, 6 and 8 weeks before entering the study among those taking OTC analgesics at least once a week in the last month?
  - How many subjects combine prescription paracetamol with OTC paracetamol or prescription NSAID with OTC NSAID?
3. Are lifestyle factors such as physical activity associated with lower prevalence of daily OTC analgesic use?

## Methods

### Study design

A cross sectional descriptive study.

### Data sources

#### Population-based health survey Nord-Trøndelag (HUNT3)

**Figure 1:**
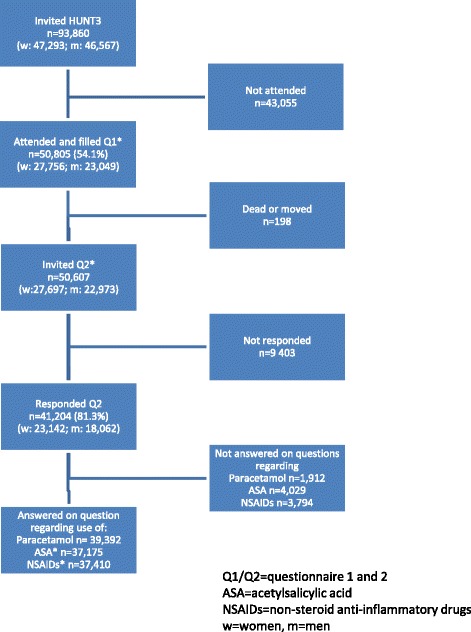
Flow chart of the study, Nord-Trøndelag Health Study (HUNT3).

All inhabitants aged 20 years or more in the county of Nord-Trøndelag in Norway were invited to participate in three population-based health surveys: the Nord-Trøndelag Health Study (HUNT) 1, 2 and 3. The HUNT Study constitutes a large population database for medical and health-related research. HUNT3 was carried out in 2006–2008, and a total of 93,860 individuals were invited to participate (Figure 1). Participants were asked to complete questionnaires. Questionnaire 1 (Q1) was mailed to them together with an invitation to a physical examination. A total of 50,805 participants returned Q1. Participants received Questionnaire 2 (Q2) at the time of the physical examination and 41,204 individuals returned Q2 by mail (Figure 1).

#### Norwegian Prescription Database (NorPD)

NorPD contains information on all prescription drugs, reimbursed or not, which are dispensed at pharmacies in Norway to individuals outside institutions [13]. Each record contains a unique person identifier, which makes it possible to identify chronologically all prescriptions to the individual subject. Prescription data are collected from pharmacies, and thus only capture prescriptions that are actually dispensed.

The two data sources HUNT3 and NorPD were linked using the unique personal identity number assigned to all individuals living in Norway.

### Questionnaires

The data used in this study were obtained from Q1 and Q2. Questionnaires are available at HUNT3 website [14]. Two questions regarding pain from Q1 were included: “Do you have bodily pain which has lasted for more than 6 months?” and “How much bodily pain have you had during the past month?” with the following response options: “None, very mild, mild, moderate, severe or very severe. Three questions addressed aspects of leisure time physical activity: The average number of times participants exercised per week, the average duration of the exercise, and average intensity of each training session”. The questions addressing exercise have shown acceptable test-retest reliability [15].

**Figure 2:**
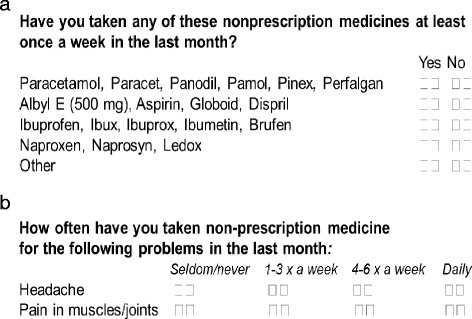
Two questions regarding use of over-the-counter (OTC) analgesics were included in Questionnaire 2 in population-based health surveys in the the Nord-Trøndelag Health Study (HUNT3). **a)** Question on type of OTC medicines and **b)** Question on frequency of use for headache pain and pain in muscle/joints.

Two questions regarding use of OTC analgesics were included in Q2 (Figure 2).

1. Question on type of OTC medicines and
2. Question on frequency of use for pain in headache and pain in muscle/joints.

Lifestyle factors of interest were selected and categorised. Categories investigated were for smoking (non-smoker, former smoker, and current smoker) and for alcohol use last 12 month (non-consumer, 0-few times, once per month-once per week, 2–7 times per week. Age at date of physical examination in the health survey was coded in 15-year categories.

### Prescription drug use

Prescribed medicines dispensed at pharmacies in Norway and recorded in NorPD are classified according to the Anatomical Therapeutic Chemical (ATC) classification system. Prescribed analgesics with the following ATC code were included in the study: paracetamol (acetaminophen) (N02BE01); acetylsalicylic acid (N02BA01), NSAIDs: ibuprofen (M01AE01) and naproxen (M01AE02). In addition, strong and weak opioids, which are classified as ATC code N02A. Opioids are only available in Norway on prescription. N02A includes all opioid analgesics marketed in Norway, but not opioids primarily used in anesthesia, substitution therapy of opioid addiction and antitussives. In Norway only physicians are allowed to prescribe analgesics.

### Definitions

Chronic pain was defined, as in previous studies, based on the HUNT 3 study: report of pain lasting six months or more and pain of at least moderate intensity during the last week prior to participation in HUNT3 [11,16].

Persistent opioid use was defined based on data from NorPD according to previously published criteria [17]. The broadest definition of persistent opioid use was applied in the present study, corresponding to use of opioids several days a week or more often. Data on dispensed opioid prescriptions during the 6 months immediately prior to participation in HUNT3 and the 12 months after the participant’s first opioid prescription were used for case definition.

### Study population

Self-reported information for all 50,805 participants from HUNT3 was linked to their prescription data from NorPD. 41,204 individuals returned Q2 by mail. Characteristics of the population who answered Q2 are available at HUNT3 webside [18]. The question regarding the use of OTC drugs in Q2 were answered by 37,175- 39,392 participants (Figure 1).

### Analysis strategy and statistical analysis

The answers from the participants regarding use of overall OTC and different OTC analgesics at least once a week in recent months (Figure 2, Tables 1 and 2) were analysed. Participants who reported use of OTC analgesics were further investigated in relation to whether they reported using these OTC drugs on a daily basis. The questions “How often have you taken non-prescription medicine for headache and/or pain in muscle/joints in the last month” were used for this purpose. In the next stage, use of OTC analgesics among participants with and without reported chronic pain was investigated. Participants with chronic pain were subsequently divided into three categories regarding use of opioids: 1) persistent users of opioids 2) non-persistent (intermittent) users of opioids and 3) individuals who were not receiving opioids.

**Table 1:** **Self-reported use of over-the-counter (OTC) analgesics (paracetamol, acetylsalicylic acid or nonsteroidal anti-inflammatory agents) at least once a week during the last month**

**All**	**Total**	**Women**	**Men**
Responders, N	39,767	22,243	17,524
Users, N (%)	18,672 (47.0)	11,708 (52.9)	6,964 (39.7)
**Chronic pain**			
Responders, N	11,761	7,252	4,508
Users, N (%)	7,543 (64.1)	5,081 (70.1)	2,462 (54.6)
**No chronic pain**			
Responders	24,398	13083	11855
Users, N (%)	9,781 (39.2)	5,708 (43.8)	4,073 (34.6)

**Table 2:** **Self-reported use of over-the-counter (OTC) analgesics at least once a week during the last month according to type of drug**

	**Paracetamol**	**Acetylsalicylic acid**	**Nonsteroidal anti-inflammatory agents (NSAIDs)**
Responders, N	39,392	37,175	37,41
Women	22,024	20,491	20,775
Men	17,368	16,684	16,635
Users, N (%)	15,093 (38.3)	3,008 (8.1)	6,978 (18.7)
Women, N (%)	9,699 (44.0)	1,398 (6.8)	4,749 (22.9)
Men, N (%)	5,394 (31.1)	1,610 (9.6)	2,229 (13.4)
Daily users*, N (%)	1,598 (10.6)	376 (12.5)	746 (10.7)
Women*, N (%)	1,203 (12.4)	246 (17.6)	560 (11.8)
Men*, N (%)	395 (7.3)	130 (8.1)	186 (8.3)
Age years, N (%)			
19-39	3,084 (39.7)	78 (1.0)	1,851 (24.0)
40-59	6,797 (40.1)	653 (4.1)	3,552 (21.6)
60-79	4,529 (35.0)	1,840 (15.4)	1,455 (12.3)
80+	683 (39.8)	437 (27.9)	120 (4.9)

Daily use is related to pain in muscles/joints and /or headache.

*% of users.

For participants who had reported use of non-prescription analgesics, analyses were performed to determine whether prescriptions of the same drug had been captured by NorPD during 4, 6 and 8 weeks prior to self-reported use.

A logistic regression was used to investigate the association between leisure time physical activity (frequency, duration, intensity) and other lifestyle factors, socio-demographic factors and health status and daily use of paracetamol and/or NSAIDs in patients with and without chronic pain. Unadjusted and adjusted odds ratios (OR) with 95% confidence intervals were calculated.

### Consent statement and ethics

All participants in HUNT3 were informed and gave consents to participation in the main and follow-up studies. All participants have consented to research being performed on their data and to linking to other data sources. The study was approved by the Regional Committee for Medical and Health Research Ethics in Central Norway. Linkage of databases was approved by the Norwegian Data Protection Authority.

## Results

### Prevalence of use of OTC analgesics

The overall prevalence of consumption of OTC analgesics at last once per week in the last month was 47% (Table 1). The prevalence was higher in those reporting chronic pain (64.1%) compared to those without chronic pain (39.2%). Women consistently reported higher prevalence compared to men.

The prevalence of self-reported use of the different OTC analgesics at last once per week in the last month was 38.3% for paracetamol, 18.7% for NSAIDs and 8.1% for ASA (Table 2). The prevalence of use of all three drug groups was higher (p < 0.001) in women compared to men. The use of NSAIDs decreased with increasing age, while ASA increased. The use of paracetamol was not influenced by age. Furthermore, about 11% of users of paracetamol, NSAIDs and ASA reported daily use of OTC analgesics for pain in muscles/joints during the last month.

### Use of OTC analgesics and reports of chronic pain

Subjects reporting chronic pain reported almost twice as high prevalence of use of paracetamol, NSAIDS and ASA (Table 3). Regardless of pain status, women (p < 0.001) used these drugs more frequently than men, except for ASA (p < 0.001).

**Table 3:** **Self-reported use of over-the-counter (OTC) analgesics at least once a week during the last month among men and women stratified on chronic pain status**

	**Paracetamol**	**Acetylsalicylic acid**	**Nonsteroidal anti-inflammatory agents (NSAIDs)**
**Chronic pain**			
Responders, N	11,548	10,452	10,619
Women	7,121	6,337	6,515
Men	4,427	4,115	4,104
Users, N	6,294	1,216	2,985
Women, N (%)*	4,322 (60.7)	638 (10.1)	2,143 (32.9)
Men, N (%)**	1,972 (44.5)	578 (14.0)	842 (20.5)
**No chronic pain**			
Responders, N	24,788	23,898	23,984
Women	13,001	12,430	12,535
Men	11,787	11,468	11,449
Users, N	7,768	1,489	3,591
Women, N (%)*	4,666 (35.9)	587 (4.7)	2,313 (18.5)
Men, N (%)**	3,102 (26.3)	902 (7.9)	1,278 (11.2)

*% of women.

**% of men.

### Use of OTC analgesics and prescription analgesics

When the study population was stratified with regard to the level of opioid use and whether they reported chronic pain or not, prevalence of OTC analgesic use was highest in persons with non-persistent (intermittent) opioid use and chronic pain (Table 4). A lower prevalence was observed in persons with persistent opioid use and chronic pain, and lowest in persons with no opioid use and chronic pain. However, the prevalence in all these three groups was approximately twice as high as in persons without chronic pain. These patterns were the same for all three groups of OTC analgesics.

**Table 4:** **Self-reported use of over-the-counter (OTC) analgesics among chronic pain patients related to their prescribed opioid use**

	**Chronic pain -persistent users of opioids**	**Chronic pain - non persistent users of opioids**	**Chronic pain- not receiving opioids**	**Non Chronic pain**
Paracetamol, N (%)	221 (63.5)	1,043 (71.5)	5,030 (51.6)	7,768 (31.3)
Acetylsalicylic acid, N (%)	35 (11.6)	206 (16.2)	975 (11.0)	1489 (6.2)
Nonsteroidal anti-inflammatory agents (NSAIDs), N (%)	91 (29.6)	457 (35.8)	2437 (27.0)	3591 (15.0)

Figures for concomitant use of OTC analgesics and prescriptions of the same group of analgesics were similar for paracetamol and NSAIDs: about 3, 4 and 5% had received prescriptions for the same group of analgesics during the last 4, 6 and 8 weeks, respectively, before HUNT 3 data collection (Table 5). 385 subjects had concomitant prescriptions for two different NSAIDS, while 440 and 196 subjects had concomitantly reported prescribed and OTC paracetamol and NSAIDs (results not shown), respectively.

**Table 5:** **Frequencies of individuals with prescription on paracetamol, acetylsalicylic acid or nonsteroidal anti-inflammatory agents (NSAIDs) among those reporting use of the same over-the-counter (OTC) analgesics during 4, 6 and 8 weeks prior to study start**

	**Self-reported use, N**	**Self-reported use + drug dispensed during 4 weeks prior to study start, N (%)**	**Self-reported use + drug dispensed during 6 weeks prior to study start, N (%)**	**Self-reported use + drug dispensed during 8 weeks prior to study start, N (%)**
Paracetamol	15,093	440 (2.9)	629 (4.2)	777 (5.1)
Acetylsalicylic acid	3,008	0 (0)	1 (0.03)	1 (0.03)
NSAIDs	6,978	196 (2.8)	278 (4.0)	366 (5.2)

### Associations between use of OTC analgesics and health/ lifestyle factors

The most important risk factor related to physical activity for using analgesics on a daily basis was exercise of short duration (Table 6). The risk (adjusted OR) was 1.5 and 4 times higher for daily use in chronic and non-chronic pain respondents in this group, respectively. Positive associations were observed between daily use of OTC analgesics and increasing age, female gender, headache and increasing pain intensity. Interestingly, consumption of alcohol 2–7 times per week reduced the risk of using OTC analgesics on a daily basis in subjects with chronic pain (OR = 0.7), compared to non-consumers.

**Table 6:** **Multivariate analysis of the association (odds ratio; OR with 95% confidence interval) between physical activity, socio demographic, lifestyle characteristics, headache, opioid use for chronic pain, pain intensity and daily use of paracetamol and/or nonsteroidal anti-inflammatory agents (NSAIDs) in persons without and with chronic pain**

	**Without chronic pain**		**Chronic pain**	
	**unadjusted OR (95% CI)**	**adjusted OR (95% CI)**	**unadjusted OR (95% CI)**	**adjusted OR (95% CI)**
**Physical activity**				
Physical activity-frequency				
non-excercise	1.3 (0.9-1.9)	0.5 (0.3-1.0)	**1.4 (1.2-1.7)**	1.0 (0.8-1.3)
once/week	1.2 (0.8-1.8)	0.6 (0.4-1.0)	1.2 (1.0-1.5)	0.9 (0.7-1.2)
2-3 times/week	0.9 (0.6-1.3)	**0.6 (0.4-1.0)**	1.1 (0.9-1.3)	1.0 (0.8-1.2)
> = 4 times /week	1 (reference)	1 (reference)	1 (reference)	1 (reference)
**Physical activity- duration**				
0-30 minutes	**4.1 (2.3-7.1)**	**4.0 (1.9-8.4)**	**2.0 (1.6.2.4)**	**1.5 (1.1-2.0)**
30-60 minutes	**2.6 (1.5-4.3)**	**2.7 (1.5-5.1)**	1.2 (1.0-1.5)	1.1 (0.9-1.4)
> = 60 minutes	1 (reference)	1 (reference)	1 (reference)	1 (reference)
**Physical activity- intensity**				
light	**6.1 (1.5-24.7)**	1.7 (0.4-7.0)	**3.0 (1.5-5.8)**	2.0 (0.9-4.5)
moderate	2.3 (0.6-9.3)	1.0 (0.2-4.3)	1.8 (0.9-3.5)	1.7 (0.8-3.7)
hard	1 (reference)	1 (reference)	1 (reference)	1 (reference)
**Gender**				
men	1 (reference)	1 (reference)	1 (reference)	1 (reference)
women	**2.4 (1.5-4.0)**	**2.4 (1.63.4)**	**1.9 (1.7-2.2)**	**1.8 (1.5-2.1)**
**Age , years**				
19-39	1 (reference)	1 (reference)	1 (reference)	1 (reference)
40-59	**2.0 (1.2-3.1)**	**2.3 (1.3-4.0)**	**1.5 (1.2-1.9)**	**1.7 (1.3-2.2)**
60-80	**3.8 (2.4-6.0)**	**5.1 (2.9-0.0)**	**2.1 (1.6-2.6)**	**2.5 (1.9-3.3)**
80+	**9.9 (5.7-17.0)**	**11.3 (5.4-23.9)**	**3.2 (2.4-4.3)**	**3.5 (2.3-5.2)**
**Smoke**				
non-smoker	1 (reference)	1 (reference)	1 (reference)	1 (reference)
former smoker	1.1 (0.8-1.5)	1.1 (0.8-1.6)	1.0 (0.9-1.1)	1.0 (0.9-1.2)
current smoker	1.2 (0.8-1.6)	1.2 (0.8-1.8)	**1.3(1.1-1.5)**	**1.4 (1.1-1.6)**
**Alcohol (frequency last 12 months)**				
non-consumer	1 (reference)	1 (reference)	1 (reference)	1 (reference)
0- few times	0.6 (0.5-1.1)	1.0 (0.5-2.0)	0.8 (0.6-1.0)	0.7 (0.5-1.0)
once per month - once per week	0.4 (0.2-0.7)	0.9 (0.5-1.8)	0.5 (0.4-0.6)	0.7 (0.5-1.0)
2-7 times per week	0.2 (0.1-0.5)	0.5 (0.3-1.2)	**0.5 (0.4-0.6)**	**0.7 (0.5-0.9)**
**Headache last 12 month**				
no	1 (reference)	1 (reference)	1 (reference)	1 (reference)
yes	**2.0 (1.5-2.6)**	**2.1 (1.5-3.0)**	**1.6 (1.4-1.8)**	**1.6 (1.4-1.8)**
**Opioid use for chronic pain**				
no use	1 (reference)	1 (reference)	1 (reference)	1 (reference)
occasional	*	*	**3.0 (3.8-6.0)**	**2.1 (1.8-2.5)**
persistent	*	*	**4.7 (3.8-6.0)**	**2.6 (1.9-3.5)**
**Pain intensity**				
no pain	1 (reference)	1 (reference)	*	
very mild	**1.9 (1.2-3.0)**	1.7 (1.0-3.1)	*	
mild	**4.9 (3.4-7.0)**	**5.1 (3.3-7.9)**	*	
moderate	**3.5 (2.3-5.5)**	**3.5 (2.0-6.0)**	1 (reference)	1 (reference)
strong	**9.4 (5.5-16.3)**	**9.4 (4.7-18.9)**	**3.0 (2.7-3.4)**	**2.5 (2.1-2.9)**
very strong	**15.4 (5.9-39.7)**	**30.1 (10.8-84.1)**	**5.0 (3.8-6.4)**	**4.8 (3.4-6.8)**

*not relevant.

number of cases in the adjusted analyses: non chronic pain n = 20 648 ; chronic pain n = 9 130.

significant results are shown in bold text.

## Discussion

This is the first study combining self-reported use of OTC analgesics from a large general population sample and prescription data from a complete national prescription database. The main finding was that the prevalence of using OTC analgesics at least once per week in the last month was 47%. Paracetamol was used by almost 40% of subjects, approximately two and four times as prevalent as NSAIDs and ASA, respectively. Among subjects reporting use of OTCs for muscle/joint pain, paracetamol, NSAIDS and ASA were consumed daily by 11% of the subjects. The use of NSAIDs decreased with age, whereas use of ASA increased in the older population. Subjects who carried out minimal physical activity, regardless of reported pain status, had 1.5-4 times greater risk of daily use of OTC. Possible concomitant use of OTC and prescribed paracetamol or NSAIDS analgesics varied between 2.8 and 5.2%.

### Prevalence compared to previous studies

Compared to previous Norwegian reports of a two-week periodic prevalence of OTC analgesic use of 28% in women and 13% in men [5] it appears that the use of OTC analgesics have increased substantially in Norway. However, even higher prevalence estimates have been reported from other countries. In a study of the US adult population from 1988 to the last month prevalence of use was 76% for non-prescription analgesics [6]. On the other hand some European studies showed lower prevalence in the use of OTC analgesics than in our study. In a Scottish study from 2005, which studied use during the preceding two weeks, a prevalence of 37% OTC analgesics was found [10]. Antonov and Isacson studied the prevalence of analgesic use in Sweden in 1998 and reported the two-week prevalence to be approximately 25% for OTC analgesics [4,9]. It must, however, be noted that direct comparison of prevalence estimates is difficult due to the differences in methodologies, recall times and case definitions.

In the present study, paracetamol was the dominating OTC drug regardless of gender and age. This was also the case for US study [6], which reported a prevalence of non-prescription analgesic use in the past month for paracetamol, ibuprofen and ASA to be around 40%, 38% and 24%. In a survey on chronic pain in European countries, use of different non-analgesics varied between countries. Non-prescription paracetamol was more frequently used in Denmark, The Netherlands, Poland, Sweden and Norway, whereas use of NSAIDs was predominant in Finland, Austria, Germany and Italy [7]. The variations are most likely attributable to cultural differences and differences in therapeutic traditions. Nevertheless, in both studies the Norwegian population had a higher prevalence of paracetamol use and a lower prevalence of NSAIDs, in particular ASA, and current practice in Norway is in line with a regulatory decision from the 1980s to switch OTC analgesic use from ASA to paracetamol for reasons of public safety.

### Gender aspect

It is generally agreed that women use more non-prescription analgesics than men and this is also confirmed at all levels in the present study [4-6,9]. This finding is probably related to findings of a higher prevalence of chronic non-malignant pain in women compared to men [19]. However, results from a Finnish study [3] did not show any obvious gender difference in analgesic use by the general population in 2002. In the Finnish study it was found a higher prevalence of daily use of both OTC analgesics and prescribed analgesics in men whereas women dominated the “a few times per week” group. This observation clearly differs from our own, in which a much higher proportion of women reported daily use of OTC analgesics, compared to men. Cultural factors may explain observed differences.

### Age

In an earlier Norwegian study [5], no relation to age was found. A Swedish study showed that individuals in the age group 18–44 years had the highest prevalence [9]. In our study, after controlling for confounding variables, a higher risk of daily use of OTC analgesics was found among older participants, both with and without chronic pain. When we studied each of the drugs used separately at least once per week in the last month, paracetamol use was age-independent, while ASA use increased with age. The use of NSAIDs decreased with age. The latter is an important observation as it complies with guidelines and recommendations asking for restrictive use of NSAIDs in the elderly population.

### Pain and opioid use

Pain intensity was a very strong predictor of OTC analgesic use in subjects with both chronic and non-chronic pain. The prevalence of OTC analgesic use was higher in persons reporting chronic pain who were non-persistent (intermittent) users of opioids, compared to those with chronic pain who were persistent users of opioids. A likely explanation is that the former group used OTC analgesics more regularly and substituted or supplemented with opioids in periods of exacerbations, while the latter did the opposite.

### Exercise and other lifestyle factors

Those subjects who reported little or no physical activity had a higher risk of using daily non-prescription analgesics than those who were active. Poor physical condition was a risk factor for analgesic use in the Swedish study [9], as well as for men only in an earlier Norwegian study [5]. Furthermore, alcohol consumption and smoking was positively related to analgesic use [9]. In the present study, smoking increased the risk of using non-prescription analgesics on a daily basis in chronic pain subjects, while frequent alcohol use had the opposite effect. It spite of no analgesic effects it can be speculated that self-medication with alcohol is a substitute for analgesic use. Differences between studies may reflect cultural and social changes over a 25- year period.

### Combination of prescription, non-prescription non-opioid analgesics

In the present study it was shown that possible concomitant use of OTC analgesics and prescribed paracetamol or NSAIDS varied between 2.8% and 5.2% depending on the duration (4, 6 or 8 weeks) of the study period. Overlapping use of both prescribed and OTC analgesics may increase the risk of interactions and overdosing [3]. Therefore physicians, when prescribing analgesics, should warn their patients against such drug combinations.

### Limitations and strengths

Strength of the present study was the use of a complete national prescription database where data are not influenced by recall bias. Another strength is the size of the population included. Subjects responded to detailed questions regarding the use of OTC drugs and a number of other potential confounders including validated questions on chronic pain and physical activity. However, the response rate was only about 50%. Even though the study population in HUNT3 is considered fairly representative of the Norwegian population, a non-responder study reported higher prevalence of musculoskeletal pain in participants compared to non-participants [20]. On the other hand a previous study of the use of opioid analgesics found that the prevalence of persistent opioid use was 1.0% percent in the HUNT3 population compared to 1.1% in the general Norwegian population, indicating that the HUNT3 participants are fairly representative with regard to analgesic use [21]. HUNT3 data were collected during 2006 to 2008 and it can be questioned whether patterns of drug use might have changed since then. However, the wholesale data have been fairly stable since 2008, indicating that findings from the HUNT3 study still have a high validity [8].

## Conclusions

The finding that 47% of those participating in the large and representative population survey used OTC analgesics at least once a week during the last month, demonstrates that such drug use is more prevalent than the self-reporting of chronic non-malignant pain of moderate or higher intensity, which in Norway is estimated to be around 30%. This indicates that mild but recurrent pain might not be detected by definitions of chronic pain in spite of leading to analgesic use. The finding that approximately 11% of those using OTC analgesics used these drugs on a daily basis raises some concerns that health care personnel need to be aware of. Long term analgesic treatment should only be commenced after a physician has considered the anticipated effect, side effects and risks of interactions. If long-term treatment is indicated the physician should prescribe these drugs instead of approving long-term OTC use. Switching to prescriptions will give the physician better control, which is in the best interest of the patient. The present findings imply that doctors should ask for OTC analgesic use, and in cases of daily use the doctor should if treatment is indicated switch to prescription use, and if not, advise to terminate or substantially reduce the OTC analgesic use.

## Abbreviations

ASA: Acetylsalicylic acid
ATC: Anatomical Therapeutic Chemical
FDA: Food and Drug Administration
HUNT: Nord–Trøndelag health study
NorPD: Norwegian Prescription Database
NSAIDs: Non-steroid anti-inflammatory drugs
OR: Odds ratios
OTC: Over-the-counter
Q1: Questionnaire 1
Q2: Questionnaire 2
